# Comparison between the Effects of Acupuncture Relative to Other Controls on Irritable Bowel Syndrome: A Meta-Analysis

**DOI:** 10.1155/2019/2871505

**Published:** 2019-11-11

**Authors:** Haizhen Zheng, Rixin Chen, Xiaofeng Zhao, Guanhui Li, Yi Liang, Hao Zhang, Zhenhai Chi

**Affiliations:** ^1^Department of Acupuncture-Moxibustion, The Affiliated Hospital of Jiangxi University of TCM, Nanchang, Jiangxi Province 330009, China; ^2^Acupuncture and Moxibustion Department, First Teaching Hospital of Tianjin University of Traditional Chinese Medicine, Tianjin 300000, China; ^3^Tianjin University of Traditional Chinese Medicine, Tianjin 300000, China

## Abstract

**Background:**

Irritable bowel syndrome (IBS) is a functional gastrointestinal disorder with recurrent abdominal pain and altered defecation habits. We here attempted to determine the effect of acupuncture on IBS.

**Methods:**

Randomized controlled trials (RCTs) published in CNKI, VIP, Wanfang, PubMed, Cochrane Library, EMBASE, Web of science, and ClinicalTrials.gov till July 17, 2019 were searched. Outcomes were total efficacy rates, overall IBS symptom scores, or global quality of life scores. Standardized mean difference (SMD) with 95% confidence intervals (CI) and risk ratio (RR) with 95% CI were calculated for meta-analysis.

**Results:**

We included 41 RCTs involving 3440 participants for analysis. 8 RCTs compared acupuncture with sham acupuncture, among which 3 trials confirmed the biological effects of acupuncture, especially in treating abdominal pain, discomfort, and stool frequency. No significant difference was found when acupuncture was compared with sham acupuncture, in terms of effects on IBS symptoms and quality of life (SMD = 0.18, 95% CI −0.26∼0.63, *P*=0.42; SMD = −0.10, 95% CI −0.31∼0.11, *P*=0.35), but the pooled efficacy rate data showed a better outcome for true acupuncture (RR = 1.22, 95% CI 1.01∼1.47, *P*=0.04), which was not supported by sensitivity analysis. Acupuncture was more effective relative to western medicine in alleviating IBS symptoms (RR = 1.17, 95% CI 1.12∼1.23, *I*^2^ = 0%, *P* < 0.00001), whose effect might last 3 months. Besides, acupuncture as an adjunct to western medicine, Chinese medications, or tuina was superior over the single latter treatment (RR = 1.68, 95% CI 1.18 to 2.40, *P*=0.004; 1.19, 1.03 to 1.36, *P*=0.02; 1.36, 1.08 to 1.72, *P*=0.009, respectively), with high heterogeneities.

**Conclusions:**

Relative to sham controls, acupuncture showed no superiority for treating IBS, while the advantage over western medicine was significant. Acupuncture could be used as an adjunct in clinical settings to improve efficacy. Future high-quality and large-sample-size studies with adequate quantity-effect design need to be conducted.

## 1. Introduction

Irritable bowel syndrome (IBS) is a functional gastrointestinal disorder with chief complaints of relapsing abdominal pain accompanied by altered bowel habits [1]. Its global prevalence was estimated up to 23% [2], and a review reported that approximately 45% children and adolescents suffered from IBS [3]. With the accelerated paces of our daily life and work, the occurrence of IBS is increasing. IBS decreases the quality of life, work productivity, and increases direct and indirect medical healthcare costs, imposing a great socioeconomic burden [4–6]. In 2008, the internationally overall medical costs regarding IBS were more than 200 billion dollars [7].

The mechanisms of IBS are still lack of understanding, to date, mainly involving the abnormality of gastrointestinal tract motility, visceral hypersensitivity, and gut microbiotic imbalance [8]. Conventional drugs including antispasmodics, fiber supplementation and antidepressants are used to alleviate the symptoms, but the limited effects are unavoidably followed by various side effects. There were about 51% of IBS patients selecting complementary and alternative medicine [9, 10], among them 59% choosing acupuncture for controlling IBS [11]. The international journals with high impacts have reported the positive effects of acupuncture on functional conditions [12, 13]. Hence, the effectiveness and better formula of acupuncture treating IBS, a functional disease, need more tests.

Earlier systematic reviews indicated the possible superiority of acupuncture over drugs, but acupuncture relative to sham acupuncture had no difference for treating IBS [14, 15]. However, sham-controlled studies on acupuncture and IBS presented inconsistent results [16–18]. Acupuncture is a comprehensive practice medicine, including body acupuncture, auricular acupuncture, scalp acupuncture, etc. The latter two belong to microsystem acupuncture based on the principle of local points reflective of the whole body situation and are quite popular in real clinical settings. So, inclusion of this kind of acupuncture is necessary to assess the true effects of acupuncture. In clinical practice, other than acupuncture, Chinese herbal medicine and tuina are also accepted by IBS patients. Owing to the respective features of the aforementioned Chinese medicine, we wanted to examine their differential effects. So as to assess the pure effect of acupuncture, we excluded the trials using acupuncture-moxibustion combined intervention, unless the control group was moxibustion to determine the add-on effect of acupuncture.

Altogether, our aim was to evaluate the differential effects of acupuncture including microsystem acupuncture relative to other controls including moxibustion and tuina therapy, for management of IBS.

## 2. Methods

### 2.1. Selection Criteria

Randomized controlled trials (RCTs) with complete baseline and valid outcome data were considered. The diagnostic criteria should be referred, including western medicine or Chinese medicine diagnostic criteria or experts opinion. The trials should compare the effects of acupuncture with other treatments for treating IBS. Acupuncture included conventional acupuncture, electro-acupuncture, and micro-puncture such as auricular acupuncture, scalp acupuncture or hand acupuncture. In order to comprehensively assess the acupuncture discipline, we included the trials with no restriction to the nationality of acupuncture. The controls should be other separate treatments other than acupuncture, such as western medicine, Chinese medicine, moxibustion, tuina, blank control, or lifestyle interventions. We also assessed the effects of acupuncture as an adjuvant to another treatment. Adjunctive treatments were allowed provided that they were given to both groups. Outcome measures should be total efficacy rates, global IBS symptom scores, overall scores of health-related quality of life. Considering the limited number of acupuncture versus sham acupuncture trials, all the related trials were retrieved. For searching clinical trials with better quality, Chinese papers we included were only published in the Chinese core journals. Graduation thesis database was also searched enabling this review to be as comprehensive as possible.

We excluded the trials using acupressure, acupoint catgut-embedding, dry needling, laser acupuncture, and percutaneous electrode nerve stimulation. To assess the sole effect of acupuncture treating IBS, we excluded the trials using co-interventions of acupuncture and moxibustion as a treatment group, but acupuncture-moxibustion versus moxibustion trials were not excluded.

### 2.2. Search Methods

Chinese databases including CNKI, VIP, and Wanfang and English databases including PubMed, Cochrane Library, EMBASE, Web of science, and ClinicalTrials.gov were searched from their inceptions to July 17, 2019. Languages were restricted to Chinese and English. For searching eligible papers as more as possible, the respective references of related trials and published systematic reviews were additionally reviewed. Searching terms were acupuncture, electroacupuncture, moxibustion, acupoint, irritable bowel syndrome, irritable bowel, irritable colon, random, or RCT. Terms combination strategies in each database were listed in [Supplementary-material supplementary-material-1].

### 2.3. Study Selection

**Figure 1:**
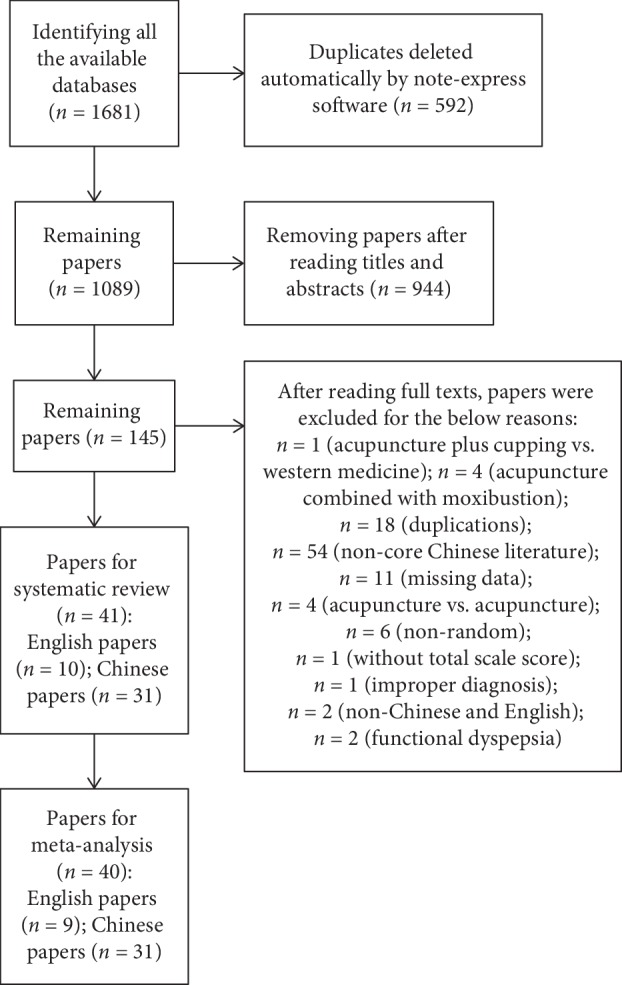
Screening flow chart.

Two authors independently selected the papers in accordance with the selection criteria. Firstly, some duplicates were excluded by the Note-express software automatically; secondly, the titles and abstracts of remaining articles were screened to exclude the obviously ineligible articles; and thirdly, the full texts of the remaining trials were downloaded and reviewed to include the finally eligible trials. The disagreements were discussed mutually and resolved by the corresponding author. The selection steps complied with the standards of Preferred Reporting Items for Systematic Reviews and Meta-Analyses (PRISMA) [19] and are presented in Figure 1.

### 2.4. Data Extraction and Data Analysis

Eligible data were extracted and analyzed by two review authors using Review Manage (RevMan) 5.3 and StataSE 12.0 software. The data included the following: general materials (titles, authors, countries, and published years), baseline characteristics (IBS diagnostic criteria, IBS type, and sample size), quality data (randomized allocation methods, allocation concealment, blinding, follow-up etc.), interventions, outcome measures, and adverse events recording. The outcomes were classified into posttreatment evaluation and follow-up evaluation according to the time points detailed in the related trials. If the eligible papers lost some necessary data, we contacted the authors via e-mails, if no reply was received, the relevant papers were excluded. Any inconsistency was resolved by a third author.

Considering the complexity of acupuncture parameters, random-effects model was used for analyzing pooled data. Because of the different evaluation scales used in the included trials, continuous data were presented in the form of standardized mean difference (SMD) and 95% confidence intervals (CI), by inverse variance method. Categorical variables were pooled as risk ratio (RR) and 95% CI, by the Mantel–Haenszel method.

In addition, we considered the dropouts in the trials as nonrespondents in accordance with the intention-to-treat (ITT) analysis principle [20], and the data in the first phase of crossover studies were retrieved. Chi-square test and I^2^ statics were used for detecting the heterogeneity across the studies, with corresponding *P* less than 0.1 and *I*^2^ ≥ 50% to indicate the significant heterogeneity [21, 22].

The risk of bias of the included studies was assessed using the guidelines from the Cochrane Collaboration's tool [23], to rate the trials as unclear risk of bias, low risk of bias, and high risk of bias. For the sham-controlled studies, if a clear blinding of the subjects was referred by the authors, or the sham acupuncture adopted Streitberger needles [24] or had been verified by pilot study, the subjects blinding was ensured. Otherwise, unclear risk of bias should be rated. The resource of other bias was defined as “baseline IBS symptom scores.”

Subgroup analysis was conducted in terms of IBS types, to judge whether the meta-analysis results would differ. Sensitivity analysis was also performed through removing the papers with unclear risk of bias, meanwhile, we included only the antispasmodics versus acupuncture data to assess the differential effects. Given that the efficacy rates were commonly used in the included trials, publication bias was detected using StataSE software to perform an Egger's test about the efficacy rates, along with a related funnel plot.

## 3. Results

### 3.1. Included Studies

Of the 1681 articles found, 41 papers were included for the systematic review and 40 for the meta-analysis. There were 31 Chinese articles, and 10 English articles. Among them, 85% were carried out by Chinese researchers; the others were by authors form United Kingdom, Germany, America, Italy, Canada, and Korea (Supplementary [Supplementary-material supplementary-material-1]). In total, 3440 participants were included in the qualitative analysis with an average of 84 persons in each trial.

### 3.2. Interventions

8 trials (16–18, 25–29) adopted sham acupuncture as the control group, among which the acupuncture total times ranged from 2 [25] to 28 [16], retention time ranged around 20–30 minutes per time, with a least acupuncture duration of 3 weeks [17, 26] and a most of 10 weeks [27]. The immediate efficacy (right at finishing treatments) of acupuncture treatments were recorded in 5 studies [16–18, 26, 28], 1 study examined acupuncture efficacy at 3 weeks post treatment [27], while 2 studies observed the efficacy at both the end of treatment and follow-up [25, 29].

23 trials compared acupuncture with western medicine [30–52]; of those, antispasmodics were used in 10 papers, antidiarrheal agents were in 2 articles, osmotic laxatives were in 4 papers, probiotics were in 6 papers, and cellulose particles in 1 study. 1 study additionally assessed the differential effects of acupuncture plus western medicine versus western medicine alone. Acupuncture treatment periods included 6 times, 9 times, 12–16 times, 18 times, 20 times, 24 times, and 28 times at most. Interventional courses ranged from 3 weeks to 6 weeks, commonly were of 4 weeks. 9 trials reported the efficacy both at the end of treatment and follow-up period, while the others simply observed the posttreatment immediate efficacy.

Acupuncture relative to Chinese herbal medicine were compared in 4 trials [32, 53–55], 4 other studies assessed the different effects of acupuncture plus Chinese medicine versus Chinese medicine [32, 56–58], acupuncture, acupuncture plus Chinese medicine versus Chinese medicine were examined in 2 studies, 3 studies selected moxibustion as a control [54, 55, 59], 1 study recorded the differential effects of acupuncture, acupuncture plus tuina, and sole tuina treatments [60]. Acupuncture times were 12 at least and 60 at most. Treatment courses were from 2 weeks to 10 weeks. Those studies only evaluated the immediate efficacy at the end of treatments.

### 3.3. Assessment of Risk of Bias

21 studies were rated as having a low risk of bias, accounting for 51% of all the included trials (Table 1). Among those non-Chinese researches, only 1 study [17] was classified into “unclear risk of bias” category, the others were into “low risk of bias” category. On the other hand, of 35 studies conducted in China, 16 studies were ranked as “low risk of bias.” Randomization and baseline IBS symptom scores items of most studies were recognized as “low risk of bias.” 11 out of 35 studies had no clear introduction of randomization methods, 30 studies were with clear randomization procedures: random number tables adopted in 17 studies, computerized randomization used in 8 trials, centralized randomization used in 3 studies, and stratified randomization in 1 trial, block randomization in 1 trial. 33 out of 41 studies had comparable baseline IBS symptom scores; the remaining 8 articles had no recording of IBS baseline symptom assessment.

Selective reporting item was assessed as low risk of bias, among which 1 study was a conference abstract, unclear risk of bias was judged as no additional information was obtained after contacting with the corresponding author, another one study providing inconsistent outcomes with the methods part was rated as high risk of bias.

Incomplete outcome data addressed were moderate. 20 studies reported withdrawals and dropouts. 5 out of 6 non-Chinese trials recorded dropouts, occupying 83%. All the dropouts were analyzed to be unrelated with the interventions.

Outcome assessor blinding was poor. 2 non-Chinese trials and 2 Chinese trials used blinding.

Patient blinding was moderate in the non-Chinese studies. 4 out of 6 studies adopted the credible sham controls: Streitberger needles used in 3 trials, penetrating needling into nonacupoints with no deqi sensation in 1 trial. Patient-blinding assessment was poor in Chinese studies, 2 studies compared acupuncture with sham acupuncture, but in view of no acupuncture reinforcing and reducing methods used in the included Chinese participants who commonly experienced acupuncture, unclear risk of bias was scored in one study; the other study used the adequate subject blinding for the detailed description of the operation. All the remaining Chinese studies were of comparative effectiveness with treatments other than acupuncture, so high risk of bias was assessed.

Allocation concealment was poor. 3 out of 6 alien studies introduced concealments, whereas only 6 out of 35 trials provided concealment procedures. 16 studies used open-label random number table methods, so this item was rated as unclear risk of bias.

## 4. Effect of Intervention

### 4.1. Acupuncture versus Sham Acupuncture

#### 4.1.1. Efficacy Rates at the End of Treatment

**Figure 2:**
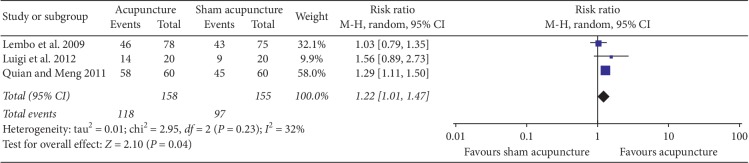
Forest plot for efficacy rates of acupuncture versus sham acupuncture at the end of treatment.

3 studies reported this item. Lembo et al. [26] included 153 patients, with whom in the acupuncture group receiving western medicine plus acupuncture (fixed formula and acupoint selection based on syndrome differentiation), whereas in the sham acupuncture group given western medicine combined with Streitberger needles stimulation at the nonacupoints near the true acupoints, twice weekly 20-minute stimulation with a total of 6 sessions over 3 weeks. One study [16] recruited 120 diarrhea-predominant IBS (D-IBS) patients, comparing acupuncture (fixed formula) with sham acupuncture at nonacupoints with no manipulations, with 28 sessions of daily 30-minute stimulation. Luigi et al. [17] randomized 40 participants into acupuncture and sham acupuncture groups, with auricular stimulation at anxiety and gut areas in the acupuncture group, and allergy areas in the placebo group, totally 6 treatments over 3 weeks. Meta-analysis showed that true acupuncture improved IBS symptoms better than sham acupuncture treatment (313 persons, RR (95% CI): 1.22 (1.01, 1.47), *I*^2^ = 32%, *P*=0.04, Figure 2).

#### 4.1.2. IBS Symptom Scores

*(1) At the End of Treatment*. Lowe et al. [25] randomized 79 subjects into true acupuncture (fixed points) and sham acupuncture groups (Streitberger needling), one 30--minute stimulation biweekly in 4 weeks. Pooled data showed no statistic difference between two groups (79 patients, SMD (95% CI): 0.18 (−0.26, 0.63), *P*=0.42, Supplementary [Supplementary-material supplementary-material-1]).

But 2 studies [16, 18] incorporated in the qualitative analysis pointed out the superiority of true acupuncture on alleviating abdominal pain, discomfort, stool frequency, and consistency.

*(1) Follow-Ups*. Forbes et al. [27] treated 59 IBS patients in the acupuncture and sham acupuncture (nonacupoints) groups, with a frequency of one time weekly totaling 10 times of treatments. IBS symptoms were analyzed at 13 weeks. Lowe et al. [25] assessed IBS symptoms at 8 weeks after the end of treatments. Pooling data found no difference between acupuncture and sham acupuncture (138 persons, SMD (95% CI): 0.21 (−0.13, 0.55), *P*=0.22, Supplementary [Supplementary-material supplementary-material-1]).

#### 4.1.3. Quality of Life Scores

*(1) At the End of Treatment*. 4 trials [25, 26, 28, 29] assessed this kind of item. Meta-analysis results indicated that no difference was found between groups (351 persons, SMD (95% CI): −0.10(−0.31, 0.11), Supplementary [Supplementary-material supplementary-material-1], *P*=0.035).

*(2) Follow-Ups*. Pooled data of 3 studies [25, 27, 29] showed the negative results (216 persons, SMD (95% CI):−0.07 (−0.34, 0.20), Supplementary [Supplementary-material supplementary-material-1], *P*=0.62).

### 4.2. Acupuncture versus Western Medicine

#### 4.2.1. Efficacy Rates

**Figure 3:**
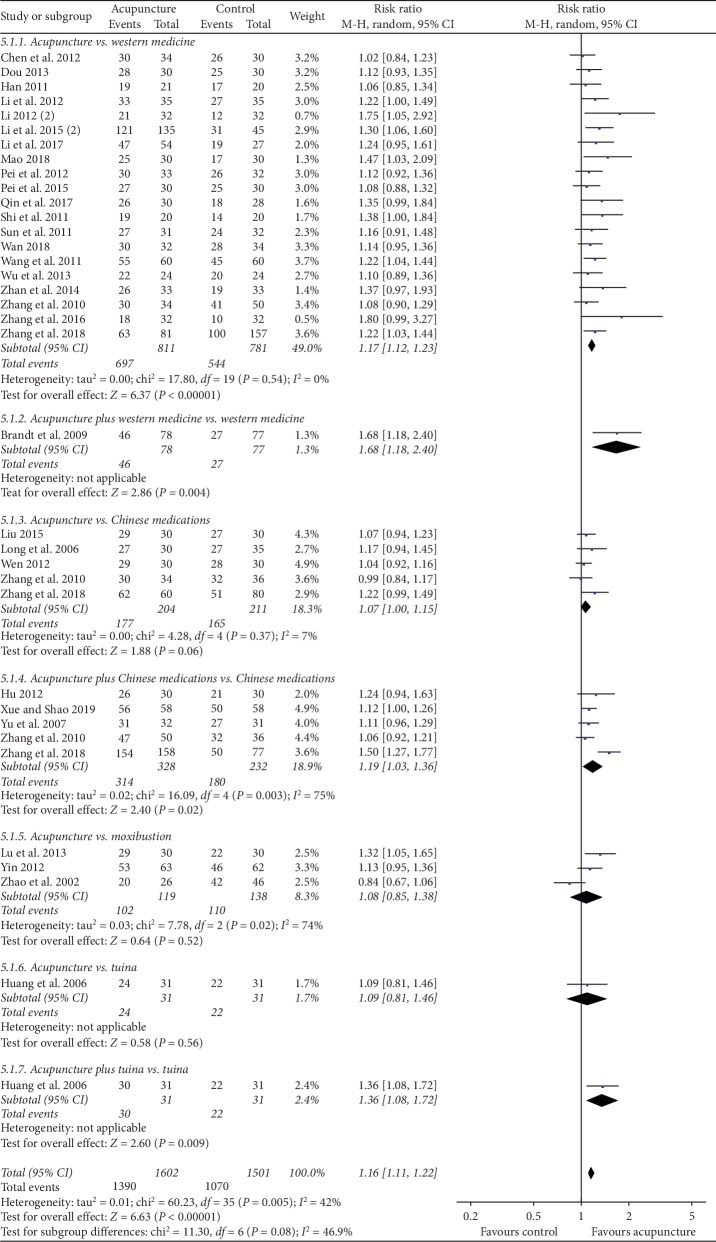
Forest plots for efficacy rates of acupuncture versus nonsham controls at the end of treatment.

*(1) At the End of Treatment*. 21 studies were included (Supplementary [Supplementary-material supplementary-material-1]), among which 9 trials adopted antispasmodics as the control, 2 trials used antidiarrheal agent, probiotics were taken in 5 studies, 4 trials offered osmotic laxatives, and cellulose was regarded as a control in 1 study. Pooled data proved the better efficacy of acupuncture than western medicine, with a low heterogeneity (1592 persons, RR (95% CI): 1.17 (1.12, 1.23), *I*^2^ = 0%, *P* < 0.00001, Figure 3).

*(2) Follow-Up Period*. In total 3 studies assessed the efficacy rate during follow-up (Supplementary [Supplementary-material supplementary-material-1]). Researchers in one study compared abdominal acupuncture, standardized acupoints, one 30-minute stimulation daily, 5 times a week, with mosapride citrate 6 mg Bid plus lactulose oral liquid 30 ml Qd, in a total of 4 weeks. At 3 months after finishing the treatments the distinct efficacy between groups was evaluated. Another study investigated the efficacy rate 3 weeks after the end of acupuncture treatment, between acupuncture (fixed acupoints, 30 minutes per session, totaling 20 times) and 50 mg Tid of pinaverium bromide for 3 weeks. 1 study assessed the effect of electro-acupuncture compared with several combination of western medicine after 4 weeks of treatment, and at week 6 of follow-up, between-group differences were statistically significant by meta-analysis (422 persons, RR (95% CI): 1.29 (1.13, 1.47), Supplementary [Supplementary-material supplementary-material-1], *P*=0.0002).

#### 4.2.2. IBS Symptom Scores

*(1) At the End of Treatment*. 14 studies (Supplementary [Supplementary-material supplementary-material-1]) reported this item. Antispasmodics were used as controls in 6 trials used cellulose, probiotics as controls in 3 studies, osmotic laxatives in 3 studies. After pooling data, IBS symptom scores were significantly decreased more by acupuncture than by western medicine, but the heterogeneity was high (1061 persons, SMD (95% CI): −1.16 (−1.61, −0.71), *I*^2^ = 91%, Supplementary [Supplementary-material supplementary-material-1], *P* < 0.00001).

*(2) Follow-Up Assessment*. Researchers in 6 papers judged the between-group symptom differences at follow-up periods. Meta-analysis supported the better improvement in acupuncture groups than controls (685 persons, SMD (95% CI): −0.76 (−1.22, −0.29), *I*^2^ = 87%, *P*=0.001, Supplementary [Supplementary-material supplementary-material-1]). Short-term follow-up (at most 3 months) had a significant improvement (SMD (95% CI): −1.02 (−1.63, −0.41), *P*=0.001, Supplementary [Supplementary-material supplementary-material-1]), however, no difference was seen during long-term follow-up (at least 6 months) (SMD (95% CI): −0.11 (−0.43, 0.21), *P*=0.49, Supplementary [Supplementary-material supplementary-material-1]).

#### 4.2.3. Quality of Life Assessment

*(1) At the End of Treatment*. 3 studies reported this item, pooling data to indicate the remarkable improvement of quality of life by acupuncture versus western medicine (190 persons, SMD (95% CI): 0.75 (0.34, 1.16), *P*=0.0003, *I*^2^ = 48%, Supplementary [Supplementary-material supplementary-material-1]).

*(2) Follow-Up*. Pooling data of 2 studies showed better quality of life by acupuncture than osmotic laxatives at 2 months after the end of treatments (120 persons, SMD (95% CI): 1.10 (0.15, 2.04), *P*=0.02, Supplementary [Supplementary-material supplementary-material-1]).

### 4.3. Acupuncture Plus Western Medicine versus Western Medicine

Meta-analysis of one non-Chinese study comparing this item proved the significance of acupuncture adjunctive to western medicine relative to western medicine (155 persons, RR (95% CI): 1.68 (1.18, 2.40), *P*=0.004, Figure 3).

### 4.4. Acupuncture versus Chinese Medicine

#### 4.4.1. Efficacy Rates

Pooling data of 5 studies found no difference between groups (415 persons, RR (95% CI): 1.07 (1.00, 1.15), *P*=0.06, Figure 3).

#### 4.4.2. IBS Symptoms

Meta-analysis of 3 researches showed significant between-group difference (280 persons, SMD (95% CI): −2.35 (−4.60, −0.09), *P*=0.04 Supplementary [Supplementary-material supplementary-material-1]).

### 4.5. Acupuncture Combined with Chinese Medicine versus Chinese Medicine

#### 4.5.1. Efficacy Rates

Acupuncture plus Chinese medicine showed better efficacy than latter alone by pooling data of 5 studies (560 persons, RR (95% CI): 1.19 (1.03, 1.36), *P*=0.02, Figure 3).

#### 4.5.2. IBS Symptoms

Meta-analysis of 2 studies showed combined groups could decrease IBS symptoms more than Chinese medication alone (295 persons, SMD (95% CI): −1.15 (−1.48, −0.81), *P* < 0.00001, Supplementary [Supplementary-material supplementary-material-1]).

#### 4.5.3. Quality of Life

Meta-analysis showed a better quality of life by combined acupuncture and Chinese medicine by one study (60 persons, SMD (95% CI): 1.57 (0.99, 2.16), *P* < 0.00001, Supplementary [Supplementary-material supplementary-material-1]).

### 4.6. Acupuncture versus Moxibustion

#### 4.6.1. Efficacy Rates

Meta-analysis of 3 related studies showed no difference between groups (257 persons, RR (95% CI): 1.08 (0.85, 1.38), *P*=0.52, Figure 3).

#### 4.6.2. IBS Symptoms

Meta-analysis of one study showed no between-group difference (60 persons, SMD (95% CI): −0.48 (−1, 0.03), *P*=0.07, Supplementary [Supplementary-material supplementary-material-1]).

### 4.7. Acupuncture versus Tuina; Acupuncture plus Tuina versus Tuina

This item was reported by 1 study showing that acupuncture relative to tuina had no superiority (62 persons, RR (95% CI): 1.09 (0.81, 1.46), *P*=0.56, Figure 3), but combination could improve the efficacy significantly (62 persons, RR (95% CI): 1.36 (1.08, 1.72), *P*=0.009, Figure 3).

## 5. Subgroup Analysis and Sensitivity Analysis

Classified by IBS types, we conducted subgroup analysis of efficacy rate between acupuncture and nonsham-acupuncture group, the results were consistent with former (Supplementary [Supplementary-material supplementary-material-1]).

After removing references having unclear risk of bias, and comparing only evidence-based antispasmodics with acupuncture, sensitivity analysis found no difference between acupuncture and sham acupuncture regarding efficacy rate, which was different from the former (Supplementary [Supplementary-material supplementary-material-1]), suggestive of invalid benefit of acupuncture compared with sham controls. Acupuncture might be more useful than moxibustion in treating constipation-type IBS (C-IBS) (Supplementary [Supplementary-material supplementary-material-1]), but more researches were necessary to further compare the distinct effects between acupuncture and moxibustion (Supplementary [Supplementary-material supplementary-material-1]).

## 6. Publication Bias Detection

Egger's test performed by StataSE software provided a *P* value of 0.649 > 0.1 (Supplementary [Supplementary-material supplementary-material-1]), showing no publication bias.

## 7. Adverse Events

11 studies [26, 28, 30–33, 37, 48, 52, 57, 62] included referred the side effects information of treatments, among which 2 studies reported a bit obvious side effects about acupuncture: 1 study [37] recording the absorbable congestion around eyes after removing needles, the other study [30] recorded one case of needle fainting which was shortly relieved by warm water. No other obvious adverse events were reported among all the studies.

## 8. Discussion

### 8.1. Summary of the Results

8 studies involved compared the differential effects of acupuncture with sham acupuncture. Of those, 7 researches were of low risk of bias, reporting withdrawals and dropouts; 5 studies used adequate random methods and participants blinding, so as to decrease the influences of subjective bias. In this systematic review, we found no significant difference when acupuncture compared with sham acupuncture, in terms of effects on IBS symptoms and quality of life, despite the pooled efficacy rate data showed a better outcome for true acupuncture, which was not supported by sensitivity analysis after removing the reference with unclear risk of bias. On the other hand, in our paper, 2 studies (a Korean and a Chinese research) indicated acupuncture could improve abdominal pain, discomforts and abnormal stool features in comparison to sham control. A paper published in JAMA showed acupuncture relative to sham acupuncture could significantly improve the urinary function [13]. Similarly, as a functional condition, IBS might benefit more from the acupoint specificity effect of true acupuncture different from sham acupuncture, so we think that the biological efficacy of acupuncture relative to sham acupuncture needs more researches of high quality to determine.

When compared with western medicine, acupuncture seemed to have a better effect regarding efficacy rates, clinical symptoms and health-related quality of life assessments, which may last 3 months. Combination of acupuncture and western medicine, larger benefits could be seen on the efficacy rates and symptoms improvements. As compared to pure Chinese medications, quality of life, and clinical symptoms could be improved better by acupuncture plus Chinese medications.

### 8.2. Limitations and Strengths

In spite of the adequate number of RCTs included in our systematic review with a relatively valid conclusion, several shortcomings should be considered with cautions: Firstly, in RCTs comparing acupuncture with sham acupuncture, the sample sizes were commonly small, except 2 trials with 120 and 153 subjects, respectively. The small sample-size studies may be not representative of the clinical effects on the patients of the same kind. Besides, the heterogeneity caused by acupuncture parameters differentials among the included studies would lead to uncertainty about the conclusion. The IBS type was also not defined within the studies, in view of the individualized specificity of acupuncture medicine principle, the true effects of acupuncture might be underestimated by the researchers, which are like 1 study [16] for D-IBS, and the positive result was obtained for acupuncture. Selecting the specific patients as the studying objects could better verify the biological effects of true acupuncture. Secondly, the risk of biases for a high proportion of RCTs in our review were ranked as unclear, which would result in operation bias to reduce the credibility of the RCTs. Thirdly, though the acupuncture measures are more than former versions, exclusion of acupressure, plastering on acupoints, percutaneous nerve electrical stimulation which were widely used in real settings would underestimate the true clinical effects of acupuncture treatments. Fourthly, a large number of included RCTs adopted some nonauthoritative indicators for efficacy like efficacy rates, a measurement without no clear and strict definition among different studies.

Among the 41 studies included in this review, 21 were literature with low risk. In 35 Chinese studies, low-risk studies take up only 46 percent, which was consistent with a former one [14]. Evaluation of acupuncture efficacy compared with western medicine, acupuncture combined with western medicine compared with western medicine, and acupuncture combined with Chinese medicine versus traditional Chinese medicine is consistent with the previous 3 systematic reviews, affirming the positive role and green characteristics of acupuncture, which is worthy of more large-sample, multicenter randomized controlled study to confirm.

Moreover, in our systematic review, we excluded the reports using co-intervention of acupuncture and moxibustion as a group, except for acupuncture plus moxibustion versus moxibustion studies, to better observe the sole effect of acupuncture. In addition, we compared acupuncture with tuina, Chinese medicine and moxibustion to mimic the real settings as far as possible, which might be valuable for clinical doctors. After all, the clinical decisions are made in a comprehensive way, including patients' preference and true facilities available. As a functional condition, the holism and variety of modalities could be necessary. Classification of the efficacy into the end of treatment and follow-up would record acupuncture effects more comprehensively. 3 sham-controlled acupuncture studies identified the acupoint specificity in the treatment of IBS, in particular for management of abdominal pain, discomfort and stool frequencies, paving a path for future confirmation.

### 8.3. Interpretation of the Results

#### 8.3.1. Acupuncture versus Sham Acupuncture

The differential results of acupuncture relative to sham acupuncture could be attributable to protocol design. At first, we could see that in RCTs supporting the superiority of acupuncture over sham controls, a Korean study [18] reported hand acupuncture versus sham control, with acupuncture parameters of twice 25-minute in a week for a total of 4 weeks. Italian researchers [17] designed an auricular stimulation as a true acupuncture group (anxiety and intestinal areas in the ears, twice weekly for 3 weeks, 6 times), while placebo acupuncture group used “allergic area” stimulation. Coming to the Chinese research [16], a fixed formula using acupuncture of daily 30 minutes' retention over 4 weeks equaling 28 sessions was compared with sham control, nonacupoints, 2 cm bilateral, perpendicular needling with no manipulation for the same course. Otherwise, the acupuncture parameters in the 5 RCTs [25–29] with negative results about the biological effects of acupuncture were not adequate in formula design. 3 studies [25, 27, 29] punctured for once a week or even once biweekly, totaling for 10 sessions or 2 times in total. Other 2 researches [26, 28] used basically enough acupuncture stimulations, relatively being twice weekly for 10 sessions over 5 weeks and twice weekly for 6 times over 3 weeks. Both studies selected credible sham control, Streitberger needles to puncture the nonacupoints nearby the true acupoints with no deqi sensation. Some researchers have recognized acupoints as depressions in an area and zone, instead of a small point. So sham acupoints bilateral away from truly classic acupoints may be still within the acupoints. Some people sensitive to acupuncture stimulation could be easy to induce a sensation identical to deqi feeling. Altogether, study design was of importance to have an impact on the effect of intervention or bluntly, adequacy of results was decided by design.

Furthermore, pooled confidence intervals for efficacy contained the possibility of better efficacy of acupuncture than sham controls. Hence, acupuncture treating IBS could be an alternative for patients. Clinical researchers showing interests in acupuncture could make a repetition of the above studies supportive of acupuncture acupoint specificity.

#### 8.3.2. Acupuncture versus Nonsham

The efficacy comparativeness studies were all undertaken in China, in which acupuncture frequencies ranged from twice weekly to once daily, total courses were between 12 times and 60 sessions, in mostly 4 weeks. Acupuncture intensity appeared larger than sham-controlled studies.

The inconsistency of the results between efficacy comparativeness and sham-controlled studies somehow could be explained by the inherent limitations of these two types of clinical studies, that is, subject blinding and between-group expectation roles were different [63–65]. The distinct expectations between medications and acupuncture could result in placebo effects differential [63, 64, 66]. Especially for this kind of functional disease, using subjective questionnaires as efficacy judgments, expectations would play a larger role which shouldn't be ignored.

Acupuncture relative to moxibustion, tuina, and Chinese herbals, treated IBS patients with inconclusive differentials due to a limited number of studies and a poor design of included studies. Further comparisons should be conducted for more choices leaving for patients.

Acupuncture as an adjunct to other interventions, including western medicine, Chinese medicine, and tuina, seemed to be promising in improvement of clinical symptoms and quality of life. Cost-effectiveness should be taken into account in making a choice for the final treatment protocol.

#### 8.3.3. Cost-Effectiveness, Safety, and Other Improvement in Acupuncture Trials for IBS

One pragmatic RCT indicated that for severer IBS patients, conventional treatments plus acupuncture could decrease the medical costs and bring more cost-effectiveness for patients [67]. In China, acupuncture has been listed in the medical insurance items, so acupuncture would be more beneficial for Chinese IBS patients, in particular in those preferring acupuncture. No serious adverse events were reported by one prospective observational study about acupuncture for IBS [68]. In our review, slightly self-absorbable blood stasis around eyes occurred after removal of needles and a case of temporary needle fainting in two studies relatively. Others found none. Credibility and expectations questionnaires should be adopted in future clinical studies to objectively analyze the biological effects of acupuncture treating IBS [69].

Experimental research has found that acupuncture might regulate brain-gut axis to alleviate IBS symptoms [70]. So in future RCTs, indicators like brain-gut peptide and inflammatory factors could be examined to better exclude the subjective impacts on acupuncture effects.

## 9. Conclusions

Taken together, for improvement of IBS symptoms and quality of life, no difference was found in acupuncture relative to sham controls studies, while some studies indicated positive results of acupuncture in alleviating IBS symptoms, reflective of a larger potential for treating IBS by true acupuncture. Acupuncture seemed to be superior over western medicine, but the placebo effects couldn't be ignored for all the studies were conducted in China. Acupuncture might be used as an adjunct to western medicine, herbal medicine and tuina for a better clinical effect. Future high-quality and large-sample studies with adequate stimulation amounts need to be conducted for further testing.

## Figures and Tables

**Table 1:** Risk of bias assessment.

Selected literature	Random method	Allocation concealment	Patient blinding	Researcher blinding	Incomplete outcome data addressed	Selective reporting	Baseline comparability	Total risk of bias
Forbes et al. [27]	Low: computer random	Low	Low: nontherapy acupoint, no arrival of qi	Low	Low	Low	Low	Low
Schneider et al. [28]	Low: center random	Low	Low: Streitberger placebo needle	Low	Low	Low	Low	Low
Lembo et al. [26]	Low: stratified random	Low	Low: Streitberger placebo needle	Unclear	Low	Low	Low	Low
Sun et al. [38]	Low: random number table	Unclear	High	Unclear	Low	Low	Low	Low
Luigi et al. [17]	Unclear	Unclear	Unclear	Unclear	Unclear	Unclear	Unclear	Unclear
Park and Cha [18]	Low: computer random	Unclear	Unclear	Unclear	Low	Low	Low	Low
Zhang et al. [32]	Low: computer random	Unclear	High	Unclear	Low	Low	Low	Low
Mak et al. [29]	Low: Block random	Low	Low: non-penetrating needle with no electrical stimulation	Low	Low	Low	Low	Low
Xue and Shao [58]	Unclear	Unclear	High	Unclear	Unclear	Low	Low	Unclear
Mao [31]	Low: random number table	Unclear	High	Unclear	Low	Low	Low	Low
Wan [30]	Low: random number table	Low	High	Unclear	Low	Low	Low	Low
Qin et al. [33]	Low: random number table	Unclear	High	Unclear	Low	Low	Low	Low
Lowe et al. [25]	Computer random	Unclear	Low: Streitberger placebo needle	Unclear	Low	Low	Low	Low
Li et al. [52]	Low: center random	Low	High	Unclear	Low	Low	Low	Low
Zhang et al. [50]	Low: random number table	Unclear	High	Unclear	Unclear	Low	Low	Unclear
Xu [51]	Low: center random	Low	High	Unclear	Unclear	Low	Low	Low
Pei et al. [47]	Low: computer random	Unclear	High	Unclear	Unclear	Low	Low	Unclear
Liu [55]	Unclear	Unclear	High	Unclear	Unclear	High	Low	Unclear
Li et al. [48]	Low: random number table	Unclear	High	Unclear	Low	Low	Unclear	Unclear
Li et al. [49]	Low: computer random	Unclear	High	Unclear	Unclear	Low	Low	Low
Zhan et al. [45]	Low: random number table	Unclear	High	Unclear	Low	Low	Low	Low
Song [46]	Low: computer random	Unclear	High	Unclear	Low	Low	Low	Low
Dou [43]	Unclear	Unclear	High	Unclear	Unclear	Low	Low	Unclear
Wu [44]	Low: random number table	Unclear	High	Unclear	Low	Low	Low	Low
Lu et al. [61]	Low: random number table	Unclear	High	Unclear	Low	Low	Low	Low
Pei et al. [39]	Low: random number table	Unclear	High	Unclear	Low	Low	Unclear	Unclear
Li et al. [40]	Low: random number table	Unclear	High	Unclear	Unclear	Low	Low	Unclear
Wen [54]	Low: random number table	Unclear	High	Unclear	Low	Low	Low	Low
Hu [57]	Unclear	Low	High	Unclear	Unclear	Low	Low	Unclear
Li [41]	Unclear	Unclear	High	Unclear	Low	Low	Low	Unclear
Chen et al. [42]	Low: random number table	Unclear	High	Unclear	Low	Low	Unclear	Unclear
Yin [62]	Low: random number table	Unclear	High	Unclear	Low	Low	Low	Low
Qian and Meng [16]	Low: computer random	Low	Unclear	Unclear	Low	Low	Low	Low
Shi et al. [35]	Unclear	Unclear	High	Unclear	Unclear	Low	Low	Unclear
Han [36]	Low: computer random	Unclear	High	Unclear	Unclear	Low	Low	Unclear
Wang et al. [37]	Low: random number table	Unclear	High	Low	Low	Low	Low	Low
Zhang et al. [34]	Unclear	Unclear	High	Unclear	Unclear	Low	Low	Unclear
Yu et al. [56]	Unclear	Unclear	High	Unclear	Unclear	Low	Unclear	Unclear
Long et al. [53]	Unclear	Unclear	High	Unclear	Low	Low	Unclear	Unclear
Huang et al. [60]	Low: random number table	Unclear	High	Unclear	Unclear	Low	Unclear	Unclear
Zhao et al. [59]	Unclear	Unclear	High	Unclear	Unclear	Low	Unclear	Unclear

^a^Baseline symptom scoring comparison.

## Supplementary Materials

Supplementary Materials Supplementary Table 1. Characteristics of selected literature on acupuncture for irritable bowel syndrome (IBS); Supplementary Table 2. Sensitivity analyses; Supplementary 1. Searching strategies; Supplementary Figure 1. Forest plot for IBS symptom scores of acupuncture versus sham acupuncture. (a) IBS symptom scores at the end of treatment; (b) IBS symptom scores at follow-ups; Supplementary Figure 2. Forest plot for quality of life scores of acupuncture versus sham acupuncture. (a) IBS-related quality of life scores at the end of treatment; (b) IBS-related quality of life scores at follow-ups; Supplementary Figure 3. Forest plot for efficacy rates of acupuncture versus western medicine at follow-ups; Supplementary Figure 4. Forest plot for IBS symptom scores of acupuncture versus nonsham control at the end of treatment; Supplementary Figure 5. Forest plot for IBS symptom scores of acupuncture versus western medicine at follow-ups; Supplementary Figure 6. Forest plot for quality of life of acupuncture versus nonsham control. a. at the end of treatment; (b) at follow-ups; Supplementary Figure 7. Forest plot for efficacy rate between acupuncture and nonsham acupuncture group-a subgroup analysis; Supplementary Figure 8. Funnel plot for publication bias detection.
