# Supplementary material for: Comparison between the Effects of Acupuncture Relative to Other Controls on Irritable Bowel Syndrome: A Meta-Analysis
Source: Pain Res Manag. 2019 Nov 11;2019:2871505. doi: 10.1155/2019/2871505 (PMC6877908; doi:10.1155/2019/2871505)
Supplement: Supplementary Materials — Supplementary Table 1. Characteristics of selected literature on acupuncture for irritable bowel syndrome (IBS); Supplementary Table 2. Sensitivity analyses; Supplementary 1. Searching strategies; Supplementary Figure 1. Forest plot for IBS symptom scores of acupuncture versus sham acupuncture. (a) IBS symptom scores at the end of treatment; (b) IBS symptom scores at follow-ups; Supplementary Figure 2. Forest plot for quality of life scores of acupuncture versus sham acupuncture. (a) IBS-related quality of life scores at the end of treatment; (b) IBS-related quality of life scores at follow-ups; Supplementary Figure 3. Forest plot for efficacy rates of acupuncture versus western medicine at follow-ups; Supplementary Figure 4. Forest plot for IBS symptom scores of acupuncture versus nonsham control at the end of treatment; Supplementary Figure 5. Forest plot for IBS symptom scores of acupuncture versus western medicine at follow-ups; Supplementary Figure 6. Forest plot for quality of life of acupuncture versus nonsham control. a. at the end of treatment; (b) at follow-ups; Supplementary Figure 7. Forest plot for efficacy rate between acupuncture and nonsham acupuncture group-a subgroup analysis; Supplementary Figure 8. Funnel plot for publication bias detection. [file 2871505.f1.zip › 2871505.f1/Supplementary Table 1.docx]

**Supplementary Table1 Characteristics of selected literature on acupuncture for irritable bowel syndrome (IBS)**

| **Author** | | **Country** | **Diagnostic criteria used for IBS** | **Types of IBS** | **Sample size** | | **Intervening measure** | | **Time point for outcome assessment** | **Assessment indicators** |
| --- | --- | --- | --- | --- | --- | --- | --- | --- | --- | --- |
|  |  |  |  |  | **Experimental**  **group** | **Control group** | **Experimental**  **group** | **Control group** |  |  |
| Acupunct-ure versus sham acupunctu-re | |  |  |  |  |  |  |  |  |  |
| Forbes  2005 ^25^ | | England | RomeⅠcriteria and Manning criteria | Irritable bowel syndrome (IBS) | 27 | 32 | selecting points according to pattern differentiation  (for up to 25min,10 sessions over 10 weeks) | sham group(not corresponding to recognized acupuncture points) | 13 weeks(follow-up period) | the symptom diary based on Bristol scale; the EuroQol quality of life questionnaire |
| Schneider2006 ^26^ | | Germany | Rome II criteria | IBS | 22 | 21 | acupuncture  (the fixed acupuncture sites, twice a week, total 10 sessions over 5 weeks) | sham acupuncture(with a blunted telescopic placebo needle that simulates an AC procedure without penetrating the skin) | 5 weeks (EoT) | quality of life questionnaire scale FDDQL |
| Lembo  2009 ^27^ | | America | Rome II criteria | IBS | 78 | 75 | medicine + acupuncture (the fixed acupuncture points and selecting points according to pattern differentiation)  20minutes per time, twice a week, for 3 weeks in total, 6sessions | medicine + sham acupuncture  (with a blunted telescopic placebo needle that simulates an AC procedure without penetrating the skin) | 3 weeks (EoT) | symptom score: IBS-AR; IBS-SSS;  quality of life score: IBS-QoL |
| Qian  2011 ^16^ | | China | Roman Ⅲ | D-  IBS | 60 | 60 | Acupuncture  (the fixed points, 30minutes per time, once a day, for 28 days in total, 28 sessions);  pinaverium bromide  (50mg,Tid,4weeks) | sham acupuncture(the fixed non-acupuncture points, 2cm, , , once a day, for 4 weeks)  pinaverium bromide(50mg,Tid,4weeks) | 4weeks(EoT) | the total efficacy rates; abdominal pain, discomforts and stool frequency scores by clinical study on new drugs of traditional Chinese medicine |
| Luigi  2012 ^17^ | | Italy | Expert/Gastroenterologist Opinion | IBS | 20 | 20 | ear acupuncture  (at the anxiety and gut area, twice a week, for 3 weeks,6 sessions) | sham ear acupuncture (in the allergy areas) | 3 weeks (EoT) | the total efficacy rates |
| Park  2012 ^18^ | | Korea | Rome III | IBS | 30 | 29 | Korean hand acupuncture(KHA)(for 25 minutes, twice a week, for 4 weeks,8 sessions) | sham KHA | 4 weeks (EoT) | Irritable bowel symptom scale |
| Lowe  2017 ^28^ | | Canada | RomeⅠ  cirteria and Manning criteria | IBS | 43 | 36 | true acupuncture  (acupuncture sites, bi-weekly for 4 weeks in 30 minute, twice in total, for 4 weeks) | sham acupuncture  (a blunt acupuncture needle, tapping against the skin at the same location as the active acupuncture site) | 4 weeks(EoT)  12weeks(8 weeks after the end of treatment) | IBS-SSS symptom scores，IBS-36 quality index |
| Mak 2019 | | China | Rome III | D-IBS | 40 | 38 | EA,30 minutes, 1 session/ week,10 weeks | Sham EA,30 minutes,blunted telescopic placebo needles hidden inside foam cubes were placed on the same acupoints as the EA group, touching the skin but without deep penetration connected with electrical wire but no electrical stimulation | 10 weeks (EoT), 6 weeks at follow-up | EuroQol 5 Dimensions to measure health-related quality of life |
| Acupunct-ure vs western medicine | |  |  |  |  |  |  |  |  |  |
| Zhang  2010 ^29^ | | China | Roman Ⅲ | D-  IBS | 34 | 50 | scalp acupuncture,5-10 minutes per time, for 4 weeks | Montmorillonite Powder, 1 bag, Tid ,for 4 weeks | 4 weeks | total efficacy rate |
| Shi2011 ^30^ | | China | RomanⅡ | IBS | 20 | 20 | Acupuncture  (fixed point, 30minutes per time, once a day, for 4weeks) | Trimebutine maleate capsule, for 2 pills ,Tid, 4 weeks | 4 weeks | mainly symptom scores; the total efficacy rate |
| Han  2011 ^31^ | | China | Roman Ⅲ | C-  IBS | 21 | 20 | acupuncture(fixed point, 30minutes per time,5 sessions a week over 4weeks) | testa triticum tricum purify, 3.5g , Tid, for 4weeks | 4 weeks(EoT),  7 weeks(the last week on a month EoT) | IBS-C clinical symptom score scales, the efficacy rate |
| Wang  2011 ^32^ | | China | Roman Ⅲ | D-  IBS | 60 | 60 | eye acupuncture(the main point + selecting points according to pattern differentiation,20minutes per time, for 4 weeks ) | pinaverium bromide  (50mg,Tid, 4weeks) | 4 weeks | the symptom scores; the efficacy rate |
| Sun  2011 ^33^ | | China | Roman Ⅲ | D-  IBS | 31 | 32 | acupuncture(the fixed point, ) | pinaverium bromide  (50mg,Tid) | 4weeks (EoT) | the total efficacy rate |
| Pei2012 ^34^ | | China | Roman Ⅲ | D-  IBS | 33 | 32 | Acupuncture  (the fixed point, 30minutes per time,5 sessions a week, over 4weeks) | pinaverium bromide  (50mg,Tid,4weeks) | 4weeks | the efficacy rate |
| Li2012 ^35^ | | China | Roman Ⅲ | D-  IBS | 35 | 35 | electro-acupuncture  (fixed point,30minutes,once a day,3-4sessions a week, over 4 weeks) | pinaverium bromide(50mg,Tid,4weeks) | 4 weeks | the symptom classification score method, the quality, the efficacy rate |
| Li2012 (2) ^36^ | | China | Roman Ⅲ | D-  IBS | 32 | 32 | Acupuncture  (fixed point, 30minutes per time,20 sessions in total) | pinaverium bromide(50mg,Tid,3 weeks) | 3weeks, 6 weeks  (3 weeks EoT) | the efficacy rate |
| Chen  2012 ^37^ | | China | Roman Ⅲ | D-  IBS | 34 | 30 | electro-acupuncture  (the fixed point,30minutes,once a day, 6sessions a week, over 4 weeks) | probiotics 0.5g, Tid, Deanxit 10.5mg,bid , for 4 weeks | 4weeks (EoT) | the total efficacy rates |
| Dou  2013 ^38^ | | China | Roman Ⅲ | C-  IBS | 30 | 30 | Acupuncture  (the fixed point, left the needle for 20 minutes, once every two days, over 4 weeks) | polyethylene glycol electrolyte dispersant,125ml per time, twice a day | 4 weeks (EoT) | traditional Chinese medicine clinical primary and secondary symptoms; the efficacy rate |
| Wu2013 ^39^ | | China | Roman Ⅲ | D-  IBS | 24 | 24 | acupuncture(the fixed point,30minutes per time, 5 sessions a week, over 4 weeks) | trigeminella bacillus Bifidobacterium,4 pills, bid , pinaverium bromide(50mg,Tid,4 weeks) | 4weeks (EoT) | traditional Chinese medicine syndrome quantization classification; the efficacy rate |
| Zhan  2014 ^40^ | | China | Roman Ⅲ | D-  IBS | 33 | 33 | acupuncture(the fixed point, 30minutes per time, 5sessions a week, over 4 weeks) | trigeminella bacillus Bifidobacterium,4 pills, Tid, pinaverium bromide  (50mg,Tid, 4 weeks) | 4 weeks(EoT) and  3 months EoT | clinical symptom classification score method, the total efficacy rate |
| Song  2014 ^41^ | | China | Roman Ⅲ | IBS | 37 | 39 | acupuncture(the fixed point,30minutes per time,5sessions a week, over 4 weeks) | trigeminella bacillus bifidobacterium,4 pills, 3x/d , pinaverium bromide tablet(50mg,3x/d,4 weeks) | 4weeks(EoT)  for 3 months、6months、9months EoT | IBS-SSS |
| Pei2015 ^42^ | | China | Roman Ⅲ | C-  IBS | 30 | 30 | electro-acupuncture (the fixed point, 30minutes a day , once a day, 5 sessions a week , 20 sessions ,over 4 weeks) | lactulose oral solution (15ml per time, Tid ,for 4 weeks) | 4 weeks (EoT) and 2 months EoT | symptom rating scale; the quality of life; the total efficacy rate |
| Li2015 ^43^ | | China | Roman Ⅲ | C-  IBS | 60 | 60 | abdominal acupuncture(the fixed point,30minutes per time, once a day,5 sessions a week , a course of the treatment for 4 weeks ) | Mosapride citrate,6mg per time, twice a day, po, lactulose oral solution,30 ml per time, once a day, po, since morning on an empty stomach, a course of treatment for 4 weeks | 4weeks(EoT)  follow-up(for 3 months EoT) | the total efficacy rate |
| Li2015 (2) ^44^ | | China | Roman Ⅲ | D-  IBS | 135 | 45 | Flexible formula;20 sessions over 4 weeks | Clostridium Butyricum Tablets(40mg,Tid)+Pinaverium Bromide Tablets(50mg,Tid);4 weeks | 4 weeks (EoT) | Total efficacy rate |
| Zhang  2016 ^45^ | | China | Roman Ⅲ | IBS | 32 | 32 | Electroacupuncture group(Fixed formula,9 sessions over 3 wks) | Trimebutine Maleate Tablets(0.2mg,Tid)+Live Combined Bifidobacterium，Lactobacillus and Enterococcus Capsules,Oral(0.42g,Bid);4 wks | 4 wks(EoT) | Total efficacy rate |
| Xu2016 ^46^ | | China | Roman Ⅲ | D-  IBS | 23 | 20 | Acupuncture group  (Fixed formula, the needles were retained for 30 min everytime,18 sessions over 6 wks) | Pinaverium Bromide Tablets(50mg,Tid,6 wks) | 6 wks (EoT) | IBS-SSS |
| Li2017 ^47^ | | China | Rome III | D-  IBS | 54 | 27 | Acupuncture group  (Fixed formula, the needles were retained for 30 min everytime,18 sessions over 6 wks) | Pinaverium Bromide Tablets  (50mg,Tid,6 wks) | 6 wks (EoT) | IBS-SSS; total efficacy rate |
| Wan2018 | | China | Roman Ⅲ | D-IBS | 32 | 34 | acupuncture, 30 minutes each time, once a day, 5 times/wk, four wks for a course of treatment, 20 times in total; EX-UE10 (*Sifeng*), 1/wk, 4 times in total | Pinaverium Bromide Tablets (50mg,Tid,4 wks) | 4 wks (EoT) | total efficacy rate |
| Mao2018 | | China | Roman Ⅲ | C-IBS | 30 | 30 | EA, 30 minutes, once a day, 5 days/wk, 4 wks, a total of 20 times | lactulose,15ml daily,5/wk,4 weeks | 4 wks (EoT), 8 wks at follow-up | IBS-C clinical symptom score, IBS-QOL, total efficacy rate |
| Zhang  2018 | | China | Roman Ⅲ | D-IBS | 81 | 157 | EA,15min, once daily, 28 sessions,4wks | compound diphenoxylate,20  mg,twice daily; montmorillonite powder,3g,  three times daily;amitriptyline,5mg,twice daily, 4 wks | 4 wks (EoT),  6 wks  at follow-up | total score for symptom severity and frequency, efficacy rate |
| Qin2017 | | China | Roman Ⅲ | D-IBS | 30 | 28 | abdominal needle,30min,3/wk,4 wks | Pinaverium Bromide Tablets (50mg,Tid,4 wks) | 4wks (EoT),  3 months at follow-up | IBS-SSS, efficacy rate |
| Acupunct-ure+west-ern medicine vs western medicine | |  |  |  |  |  |  |  |  |  |
| Lembo  2009 ^27^ | | America | Rome II | IBS | 78 | 77 | Medicine+Acupuncture  (Fixed formula+flexible formula,the needles were left in place for 20 min everytime,6 sessions over 3 wks) | medicine | 3 wks (EoT) | IBS-AR, IBS-SSS, IBS-QoL |
| Acupunct-ure vs traditional Chinese medicine | |  |  |  |  |  |  |  |  |  |
| Long  2006 ^48^ | | China | RomanⅡ | C-  IBS | 30 | 35 | Acupuncture  (the needles were retained for 30 min everytime,14 sessions over 2 wks)+Live Bifidobacterium Preparation，Oral(4 pills, Tid, 2 wks) | Tong Bian Ling Capsule(2 pills  ,Bid to Tid,5-7d)+Live Bifidobacterium Preparation，Oral  (4 pills,Tid,2 wks) | 2 wks | Efficacy rate |
| Zhang  2010 ^29^ | | China | Roman Ⅲ | D-  IBS | 34 | 36 | Scalp acupuncture group,the needles were left in place for 5-10min everytime,4wks | Chinese herbal medicine group, one dose a day, 4 wks | 4 wks(EoT) | Total efficacy rate |
| Wen  2012 ^49^ | | China | Roman Ⅲ | D-  IBS | 30 | 30 | Acupuncture group(Fixed formula+flexible formula,the needles were left in place for 20 min everytime,15 sessions over 8 wks) | Tongxieyao formula,2g,Tid,4wks | EoT | Bowel sympotom scale total scores;  Total efficacy rate |
| Liu2015 ^50^ | | China | Roman Ⅲ | D-  IBS | 30 | 30 | Acupuncture group(  Fixed formula,the needles were left in place for 30 min everytime,20 sessions over 20d) | Jia Wei Xiao Yao Wan(1bag/6g,2×/d,20d) | 20 d (EoT) | Diarrhea symptom score; Total efficacy rate |
| Zhang  2018 | | China | Roman Ⅲ | D-  IBS | 80 | 80 | EA,15 min, once daily, 28 sessions, 4wks | Qibei mixture group, 6g, twice daily, 4 wks | 4 wks (EoT), 6 wks at follow-up | total score for symptom severity and frequency, efficacy rate |
| Acupuncture+ traditional Chinese medicine vs traditional Chinese medicine | | |  |  |  |  |  |  |  |  |
| Yu  2007 ^51^ | | China | The diagnostic Criteria for Irritable Bowel Syndrome proposed on National Symposium on Chronic Non- Infectious Intestinal Diseases In June 1993 | D-  IBS | 32 | 31 | Fixed formula with Acupuncture ,the needles were left in place for 30 min everytime,28 sessions over 4 wks+Chinese herbal formula(6 pills,3×/d,4 wks) | Chinese herbal formula alone | 4 wks(EoT) | Efficacy rate |
| Zhang  2010 ^29^ | | China | Roman Ⅲ | D-  IBS | 50 | 36 | Scalp acupuncture,the needles were left in place for 5-10min everytime,4wks+Chinese herbal medicine,one dose a day,4 wks | Chinese herbal medicine,one dose a day,4 wks | 4 wks(EoT) | Total efficacy rate |
| Hu  2012 ^52^ | | Chian | Roman Ⅲ | IBS | 30 | 30 | Electroacupuncture  (the needles were left in place for 30min everytime,20 sessions over 4 wks)+Additive ShenLingBaiZhu Decoction(one dose a day,20 sessions over 4 wks) | Additive ShenLingBaiZhu Decoction (one dose a day,20 sessions over 4 wks) | 1 mos (EoT) | Symptoms score; QoL;  Efficacy rate |
| Zhang  2018 | | China | Roman Ⅲ | D-  IBS | 158 | 77 | EA,15 min, once daily, 28 sessions, 4wks; Qibei mixture group, 6g, twice daily, 4 wks | Qibei mixture group, 6g, twice daily, 4 wks | 4 wks (EoT), 6 wks at follow-up | total score for symptom severity and frequency, efficacy rate |
| Xue 2019 | | China | Roman Ⅲ | D-  IBS | 58 | 58 | acupuncture therapy of TCM on the condition of the control group, 30 minutes per time, 5 times/week, for 1 month | 20-40mL of Astragalus injection was mixed with 250–500 mL of normal saline or glucose for IV infusion of children once a day，1 month | 1 month (EoT) | efficiency rate |
| Acupunct-ure vs moxibustion | |  |  |  |  |  |  |  |  |  |
| Zhao  2002 ^53^ | | China | 1992 Roman | D-  IBS | 26 | 46 | Acupuncture  (Fixed formula with ,24 sessions over 8 wks) | Moxibustion(Drug-separated Moxibustion,1-2 zhuang each time,24 sessions over 8 wks) | 8 wks | Total efficacy rate |
| Yin  2012 ^54^ | | China | Roman Ⅲ | C-  IBS and D-  IBS | 32（D-IBS）; 31（C-IBS） | 30（D-IBS）；32（C-IBS） | Electroacupuncture group (Fixed formula,the needles were left in place for 30min everytime,24 sessions over 4 wks) | Suspended moxibustion group (30 min,24 sessions over 4 wks) | 4 wks(EoT) | Total efficacy rate |
| Lu  2013 ^55^ | | China | Roman Ⅲ | C-  IBS | 30  30 (the number after attrition ) | 30  (the number after attrition ) | Electroacupuncture group(Fixed formula,the needles were left in place for 20 min everytime,12 sessions over 4 wks) | Suspended moxibustion group(30-40 min,12 sessions over 4 wks) | 4 wks(EoT) | Bristol Symptom Scale;Total efficacy rate |
| Acupunct-ure vs tuina | |  |  |  |  |  |  |  |  |  |
| Huang  2006 ^56^ | | China | RomanⅡ | IBS | 31 | 31 | Acupuncture group  (the needles were left in place for 30 min everytime,once daily,60 sessions over 70d) | Tuina (once daily,v60 sessions over 70d) | 70d (EoT) | Total efficacy rate |
| Acupuncture + tuina  vs tuina | | |  |  |  |  |  |  |  |  |
| Huang2006 ^56^ | China | | RomanⅡ | IBS | 31 | 31 | Acupuncture combined with tuina (acupuncture for 30 min,tuina for 10 min,once daily,60 sessions over 70d) | Tuina (once daily,60 sessions over 70d) | 70d (EoT) | Total efficacy rate |
