# Supplementary figures and images for: Comparison between the Effects of Acupuncture Relative to Other Controls on Irritable Bowel Syndrome: A Meta-Analysis
Source: Pain Res Manag. 2019 Nov 11;2019:2871505. doi: 10.1155/2019/2871505 (PMC6877908; doi:10.1155/2019/2871505)

**a**

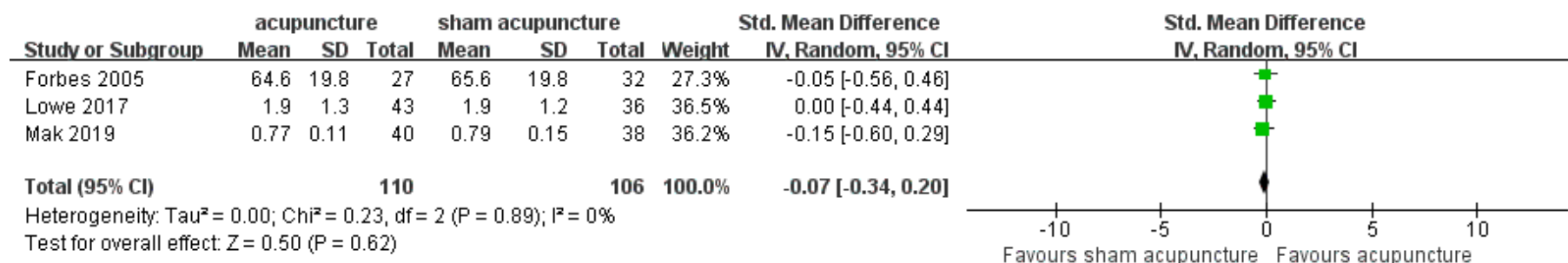

**b**

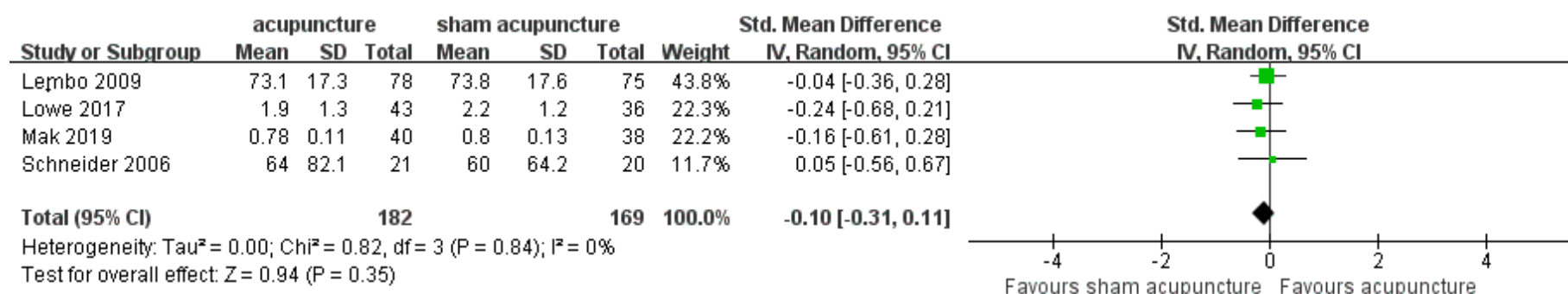

Supplement: Supplementary Materials — Supplementary Table 1. Characteristics of selected literature on acupuncture for irritable bowel syndrome (IBS); Supplementary Table 2. Sensitivity analyses; Supplementary 1. Searching strategies; Supplementary Figure 1. Forest plot for IBS symptom scores of acupuncture versus sham acupuncture. (a) IBS symptom scores at the end of treatment; (b) IBS symptom scores at follow-ups; Supplementary Figure 2. Forest plot for quality of life scores of acupuncture versus sham acupuncture. (a) IBS-related quality of life scores at the end of treatment; (b) IBS-related quality of life scores at follow-ups; Supplementary Figure 3. Forest plot for efficacy rates of acupuncture versus western medicine at follow-ups; Supplementary Figure 4. Forest plot for IBS symptom scores of acupuncture versus nonsham control at the end of treatment; Supplementary Figure 5. Forest plot for IBS symptom scores of acupuncture versus western medicine at follow-ups; Supplementary Figure 6. Forest plot for quality of life of acupuncture versus nonsham control. a. at the end of treatment; (b) at follow-ups; Supplementary Figure 7. Forest plot for efficacy rate between acupuncture and nonsham acupuncture group-a subgroup analysis; Supplementary Figure 8. Funnel plot for publication bias detection. [file 2871505.f1.zip › 2871505.f1/Supplementary Fig 2.pdf]

**a**

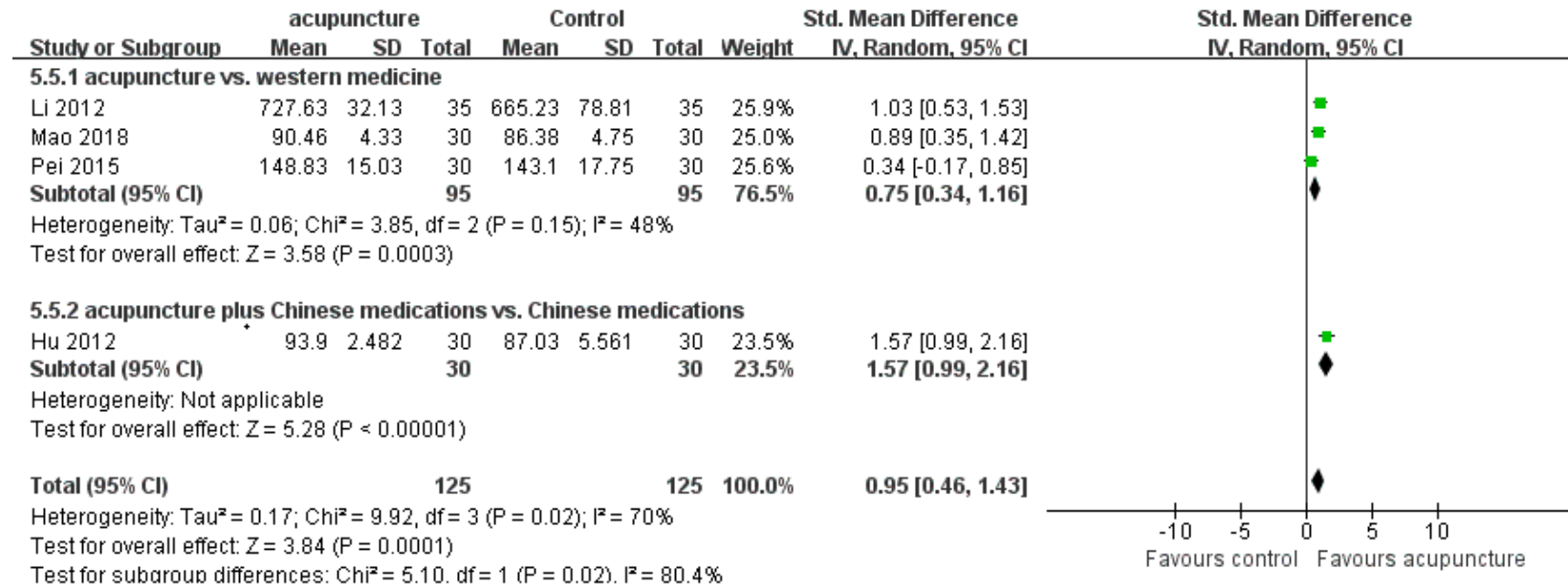

**b**

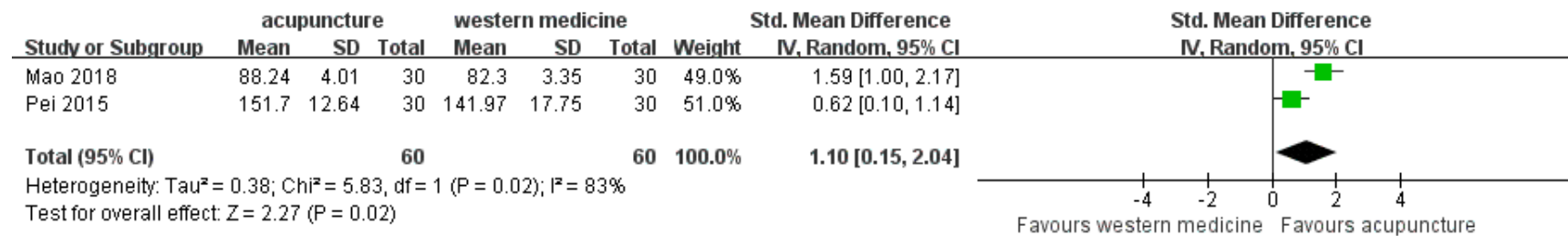

Supplement: Supplementary Materials — Supplementary Table 1. Characteristics of selected literature on acupuncture for irritable bowel syndrome (IBS); Supplementary Table 2. Sensitivity analyses; Supplementary 1. Searching strategies; Supplementary Figure 1. Forest plot for IBS symptom scores of acupuncture versus sham acupuncture. (a) IBS symptom scores at the end of treatment; (b) IBS symptom scores at follow-ups; Supplementary Figure 2. Forest plot for quality of life scores of acupuncture versus sham acupuncture. (a) IBS-related quality of life scores at the end of treatment; (b) IBS-related quality of life scores at follow-ups; Supplementary Figure 3. Forest plot for efficacy rates of acupuncture versus western medicine at follow-ups; Supplementary Figure 4. Forest plot for IBS symptom scores of acupuncture versus nonsham control at the end of treatment; Supplementary Figure 5. Forest plot for IBS symptom scores of acupuncture versus western medicine at follow-ups; Supplementary Figure 6. Forest plot for quality of life of acupuncture versus nonsham control. a. at the end of treatment; (b) at follow-ups; Supplementary Figure 7. Forest plot for efficacy rate between acupuncture and nonsham acupuncture group-a subgroup analysis; Supplementary Figure 8. Funnel plot for publication bias detection. [file 2871505.f1.zip › 2871505.f1/Supplementary Fig 6.pdf]

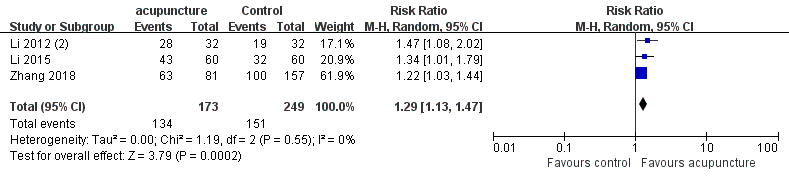

Supplement: Supplementary Materials — Supplementary Table 1. Characteristics of selected literature on acupuncture for irritable bowel syndrome (IBS); Supplementary Table 2. Sensitivity analyses; Supplementary 1. Searching strategies; Supplementary Figure 1. Forest plot for IBS symptom scores of acupuncture versus sham acupuncture. (a) IBS symptom scores at the end of treatment; (b) IBS symptom scores at follow-ups; Supplementary Figure 2. Forest plot for quality of life scores of acupuncture versus sham acupuncture. (a) IBS-related quality of life scores at the end of treatment; (b) IBS-related quality of life scores at follow-ups; Supplementary Figure 3. Forest plot for efficacy rates of acupuncture versus western medicine at follow-ups; Supplementary Figure 4. Forest plot for IBS symptom scores of acupuncture versus nonsham control at the end of treatment; Supplementary Figure 5. Forest plot for IBS symptom scores of acupuncture versus western medicine at follow-ups; Supplementary Figure 6. Forest plot for quality of life of acupuncture versus nonsham control. a. at the end of treatment; (b) at follow-ups; Supplementary Figure 7. Forest plot for efficacy rate between acupuncture and nonsham acupuncture group-a subgroup analysis; Supplementary Figure 8. Funnel plot for publication bias detection. [file 2871505.f1.zip › 2871505.f1/Supplementary Fig. 3.tif]

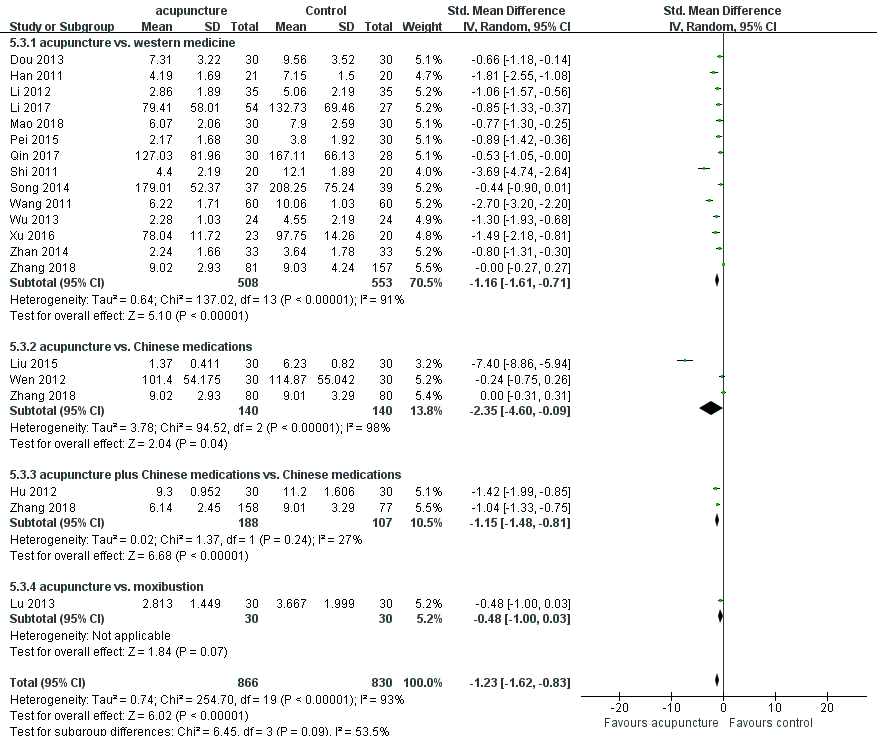

Supplement: Supplementary Materials — Supplementary Table 1. Characteristics of selected literature on acupuncture for irritable bowel syndrome (IBS); Supplementary Table 2. Sensitivity analyses; Supplementary 1. Searching strategies; Supplementary Figure 1. Forest plot for IBS symptom scores of acupuncture versus sham acupuncture. (a) IBS symptom scores at the end of treatment; (b) IBS symptom scores at follow-ups; Supplementary Figure 2. Forest plot for quality of life scores of acupuncture versus sham acupuncture. (a) IBS-related quality of life scores at the end of treatment; (b) IBS-related quality of life scores at follow-ups; Supplementary Figure 3. Forest plot for efficacy rates of acupuncture versus western medicine at follow-ups; Supplementary Figure 4. Forest plot for IBS symptom scores of acupuncture versus nonsham control at the end of treatment; Supplementary Figure 5. Forest plot for IBS symptom scores of acupuncture versus western medicine at follow-ups; Supplementary Figure 6. Forest plot for quality of life of acupuncture versus nonsham control. a. at the end of treatment; (b) at follow-ups; Supplementary Figure 7. Forest plot for efficacy rate between acupuncture and nonsham acupuncture group-a subgroup analysis; Supplementary Figure 8. Funnel plot for publication bias detection. [file 2871505.f1.zip › 2871505.f1/Supplementary Fig. 4.tif]

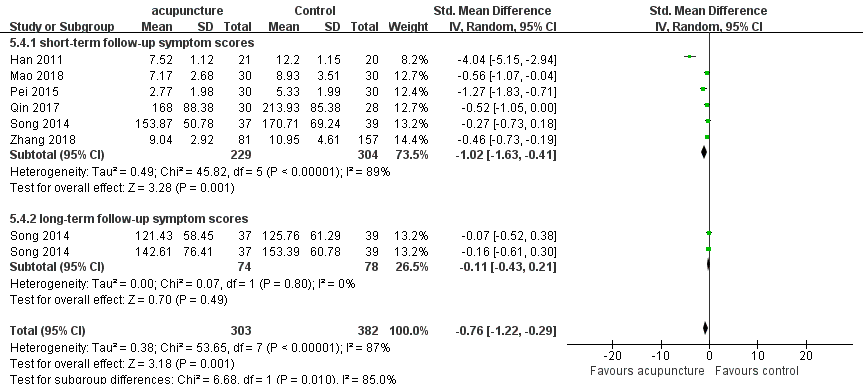

Supplement: Supplementary Materials — Supplementary Table 1. Characteristics of selected literature on acupuncture for irritable bowel syndrome (IBS); Supplementary Table 2. Sensitivity analyses; Supplementary 1. Searching strategies; Supplementary Figure 1. Forest plot for IBS symptom scores of acupuncture versus sham acupuncture. (a) IBS symptom scores at the end of treatment; (b) IBS symptom scores at follow-ups; Supplementary Figure 2. Forest plot for quality of life scores of acupuncture versus sham acupuncture. (a) IBS-related quality of life scores at the end of treatment; (b) IBS-related quality of life scores at follow-ups; Supplementary Figure 3. Forest plot for efficacy rates of acupuncture versus western medicine at follow-ups; Supplementary Figure 4. Forest plot for IBS symptom scores of acupuncture versus nonsham control at the end of treatment; Supplementary Figure 5. Forest plot for IBS symptom scores of acupuncture versus western medicine at follow-ups; Supplementary Figure 6. Forest plot for quality of life of acupuncture versus nonsham control. a. at the end of treatment; (b) at follow-ups; Supplementary Figure 7. Forest plot for efficacy rate between acupuncture and nonsham acupuncture group-a subgroup analysis; Supplementary Figure 8. Funnel plot for publication bias detection. [file 2871505.f1.zip › 2871505.f1/Supplementary Fig. 5.tif]

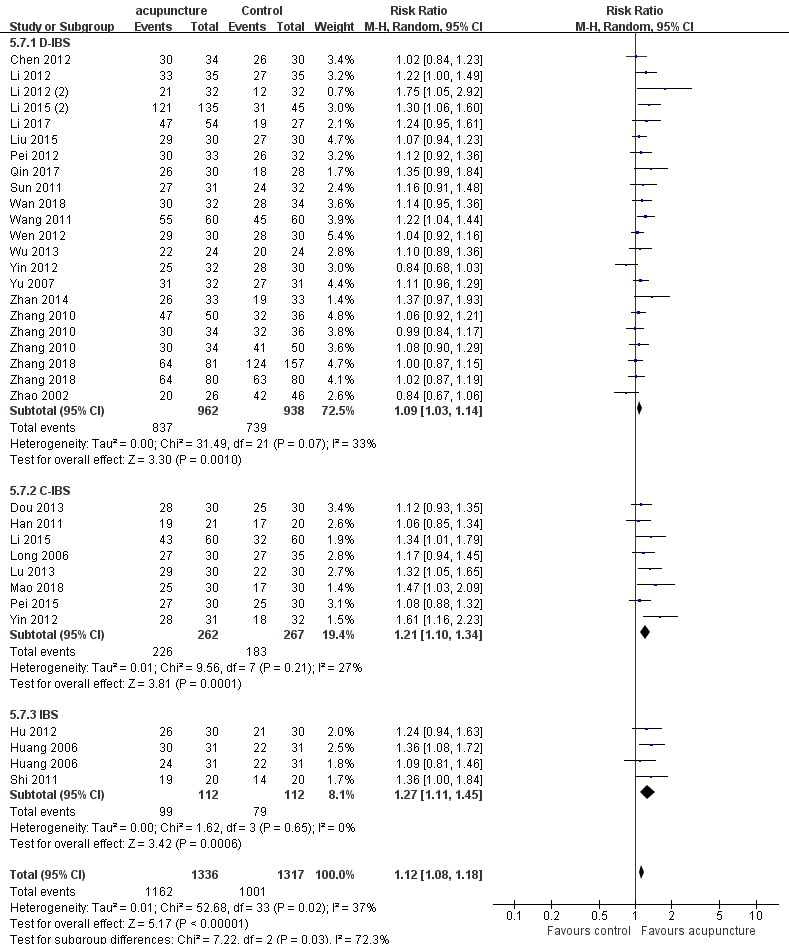

Supplement: Supplementary Materials — Supplementary Table 1. Characteristics of selected literature on acupuncture for irritable bowel syndrome (IBS); Supplementary Table 2. Sensitivity analyses; Supplementary 1. Searching strategies; Supplementary Figure 1. Forest plot for IBS symptom scores of acupuncture versus sham acupuncture. (a) IBS symptom scores at the end of treatment; (b) IBS symptom scores at follow-ups; Supplementary Figure 2. Forest plot for quality of life scores of acupuncture versus sham acupuncture. (a) IBS-related quality of life scores at the end of treatment; (b) IBS-related quality of life scores at follow-ups; Supplementary Figure 3. Forest plot for efficacy rates of acupuncture versus western medicine at follow-ups; Supplementary Figure 4. Forest plot for IBS symptom scores of acupuncture versus nonsham control at the end of treatment; Supplementary Figure 5. Forest plot for IBS symptom scores of acupuncture versus western medicine at follow-ups; Supplementary Figure 6. Forest plot for quality of life of acupuncture versus nonsham control. a. at the end of treatment; (b) at follow-ups; Supplementary Figure 7. Forest plot for efficacy rate between acupuncture and nonsham acupuncture group-a subgroup analysis; Supplementary Figure 8. Funnel plot for publication bias detection. [file 2871505.f1.zip › 2871505.f1/Supplementary Fig. 7.tif]

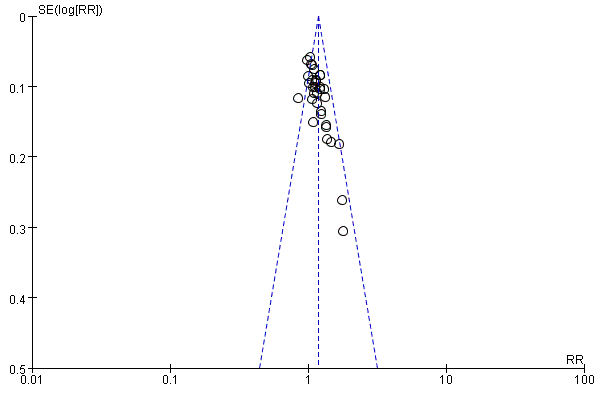

Supplement: Supplementary Materials — Supplementary Table 1. Characteristics of selected literature on acupuncture for irritable bowel syndrome (IBS); Supplementary Table 2. Sensitivity analyses; Supplementary 1. Searching strategies; Supplementary Figure 1. Forest plot for IBS symptom scores of acupuncture versus sham acupuncture. (a) IBS symptom scores at the end of treatment; (b) IBS symptom scores at follow-ups; Supplementary Figure 2. Forest plot for quality of life scores of acupuncture versus sham acupuncture. (a) IBS-related quality of life scores at the end of treatment; (b) IBS-related quality of life scores at follow-ups; Supplementary Figure 3. Forest plot for efficacy rates of acupuncture versus western medicine at follow-ups; Supplementary Figure 4. Forest plot for IBS symptom scores of acupuncture versus nonsham control at the end of treatment; Supplementary Figure 5. Forest plot for IBS symptom scores of acupuncture versus western medicine at follow-ups; Supplementary Figure 6. Forest plot for quality of life of acupuncture versus nonsham control. a. at the end of treatment; (b) at follow-ups; Supplementary Figure 7. Forest plot for efficacy rate between acupuncture and nonsham acupuncture group-a subgroup analysis; Supplementary Figure 8. Funnel plot for publication bias detection. [file 2871505.f1.zip › 2871505.f1/Supplementary Fig. 8. Funnel plot for publication bias detection.tif]

**a**

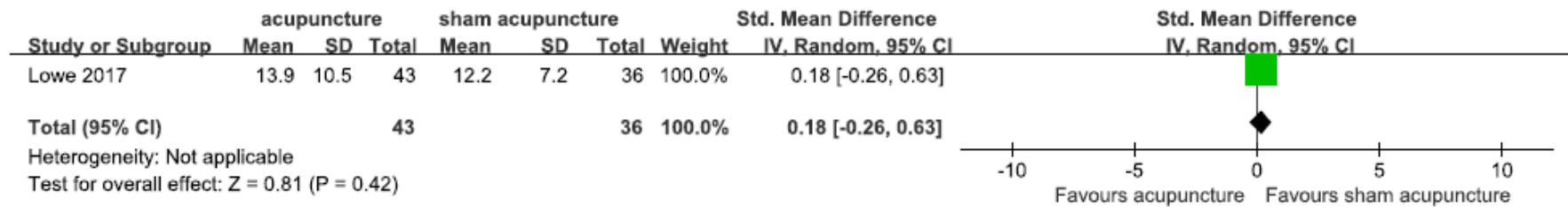

**b**

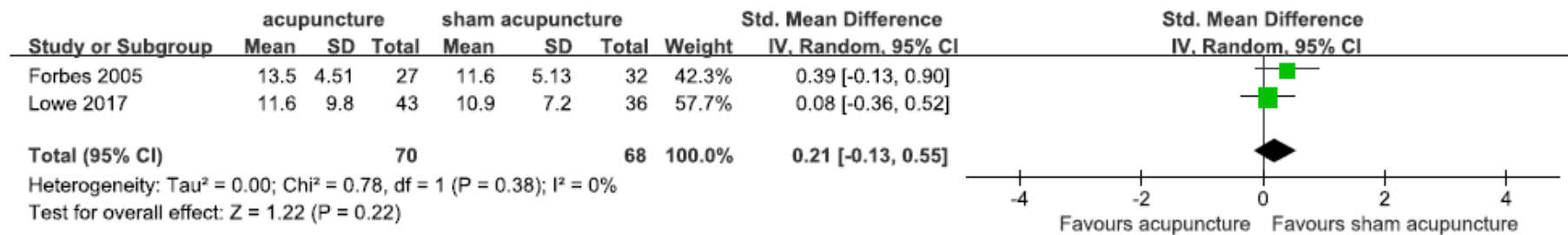

Supplement: Supplementary Materials — Supplementary Table 1. Characteristics of selected literature on acupuncture for irritable bowel syndrome (IBS); Supplementary Table 2. Sensitivity analyses; Supplementary 1. Searching strategies; Supplementary Figure 1. Forest plot for IBS symptom scores of acupuncture versus sham acupuncture. (a) IBS symptom scores at the end of treatment; (b) IBS symptom scores at follow-ups; Supplementary Figure 2. Forest plot for quality of life scores of acupuncture versus sham acupuncture. (a) IBS-related quality of life scores at the end of treatment; (b) IBS-related quality of life scores at follow-ups; Supplementary Figure 3. Forest plot for efficacy rates of acupuncture versus western medicine at follow-ups; Supplementary Figure 4. Forest plot for IBS symptom scores of acupuncture versus nonsham control at the end of treatment; Supplementary Figure 5. Forest plot for IBS symptom scores of acupuncture versus western medicine at follow-ups; Supplementary Figure 6. Forest plot for quality of life of acupuncture versus nonsham control. a. at the end of treatment; (b) at follow-ups; Supplementary Figure 7. Forest plot for efficacy rate between acupuncture and nonsham acupuncture group-a subgroup analysis; Supplementary Figure 8. Funnel plot for publication bias detection. [file 2871505.f1.zip › 2871505.f1/Supplementary Figure 1.pdf]
