# Supplementary material for: Comparison between the Effects of Acupuncture Relative to Other Controls on Irritable Bowel Syndrome: A Meta-Analysis
Source: Pain Res Manag. 2019 Nov 11;2019:2871505. doi: 10.1155/2019/2871505 (PMC6877908; doi:10.1155/2019/2871505)
Supplement: Supplementary Materials — Supplementary Table 1. Characteristics of selected literature on acupuncture for irritable bowel syndrome (IBS); Supplementary Table 2. Sensitivity analyses; Supplementary 1. Searching strategies; Supplementary Figure 1. Forest plot for IBS symptom scores of acupuncture versus sham acupuncture. (a) IBS symptom scores at the end of treatment; (b) IBS symptom scores at follow-ups; Supplementary Figure 2. Forest plot for quality of life scores of acupuncture versus sham acupuncture. (a) IBS-related quality of life scores at the end of treatment; (b) IBS-related quality of life scores at follow-ups; Supplementary Figure 3. Forest plot for efficacy rates of acupuncture versus western medicine at follow-ups; Supplementary Figure 4. Forest plot for IBS symptom scores of acupuncture versus nonsham control at the end of treatment; Supplementary Figure 5. Forest plot for IBS symptom scores of acupuncture versus western medicine at follow-ups; Supplementary Figure 6. Forest plot for quality of life of acupuncture versus nonsham control. a. at the end of treatment; (b) at follow-ups; Supplementary Figure 7. Forest plot for efficacy rate between acupuncture and nonsham acupuncture group-a subgroup analysis; Supplementary Figure 8. Funnel plot for publication bias detection. [file 2871505.f1.zip › 2871505.f1/Supplementary 1.doc]

**Searching strategies**

**1. PubMed database: from inception to July 17, 2019.**

 ((((((((irritable bowel syndrome[MeSH Terms]) OR Irritable Bowel Syndromes) OR Syndrome, Irritable Bowel) OR Syndromes, Irritable Bowel) OR Colon, Irritable) OR Irritable Colon)) AND ((((((((Acupuncture[MeSH Terms]) OR Acupoint*) OR auriculoacupuncture) OR Electroacupuncture) OR Electrical) OR Electric) OR Electrotherapy) OR Electro-acupuncture)) AND (((randomized controlled trial[Publication Type]) OR clinical trial[Publication Type]) OR clinical study[Publication Type])

**2. EMBASE database: from inception to July 17, 2019.**

'irritable colon'/exp OR irritable AND ('colon'/exp OR colon) OR 'short bowel syndrome'/exp OR short AND ('bowel'/exp OR bowel) AND ('syndrome'/exp OR syndrome) AND ('acupuncture'/exp OR acupuncture OR 'electroacupuncture'/exp OR electroacupuncture) AND (randomized OR trial)

**3. Web of Science: from inception to July 17, 2019.**

TS= (Irritable bowel syndrome OR irritable Colon) AND TS=(acupoint* OR *acupuncture OR electric OR electrical OR electrotherapy) AND TS=(random* AND (trial* OR study))

**4. Cochrane Library:** **from inception to July 17, 2019.**

(Irritable Bowel Syndrome*:ti,ab,kw or irritable colon:ti,ab,kw or Neurogenic Bowel:ti,ab,kw or Colonic Diseases, Functional:ti,ab,kw or Colonic Pseudo-Obstruction:ti,ab,kw) AND (acupoint* or *acupuncture or electric or electrical or electrotherapy:ti,ab,kw) AND (random* and (trial* or study):ab)

**5. ClinicalTrials.gov: from inception to July 17, 2019.**

(“Functional bowel disease” OR “Irritable Colon” OR “Irritable bowel” OR “Nervous colitis” OR “bowel syndrome irritable” OR “bowels irritable” OR “colon irritable” OR “functional bowel syndrome”) AND (“acupuncture” OR “electroacupuncture” OR “electro-acupuncture” OR “electric”)

**6. CNKI: from inception to July 17, 2019.**

(SU=acupuncture or needling) AND (TI=irritable bowel syndrome) AND (FT=random OR clinical)

**7. Wanfang database: from inception to July 17, 2019.**

(SU=acupuncture or needling) AND (TI=irritable bowel syndrome) AND (FT=random OR clinical)

**8. VIP: from inception to July 17, 2019.**

(SU=acupuncture or needling) AND (TI=irritable bowel syndrome) AND (FT=random OR clinical)
