# Supplementary material for: Comparison between the Effects of Acupuncture Relative to Other Controls on Irritable Bowel Syndrome: A Meta-Analysis
Source: Pain Res Manag. 2019 Nov 11;2019:2871505. doi: 10.1155/2019/2871505 (PMC6877908; doi:10.1155/2019/2871505)
Supplement: Supplementary Materials — Supplementary Table 1. Characteristics of selected literature on acupuncture for irritable bowel syndrome (IBS); Supplementary Table 2. Sensitivity analyses; Supplementary 1. Searching strategies; Supplementary Figure 1. Forest plot for IBS symptom scores of acupuncture versus sham acupuncture. (a) IBS symptom scores at the end of treatment; (b) IBS symptom scores at follow-ups; Supplementary Figure 2. Forest plot for quality of life scores of acupuncture versus sham acupuncture. (a) IBS-related quality of life scores at the end of treatment; (b) IBS-related quality of life scores at follow-ups; Supplementary Figure 3. Forest plot for efficacy rates of acupuncture versus western medicine at follow-ups; Supplementary Figure 4. Forest plot for IBS symptom scores of acupuncture versus nonsham control at the end of treatment; Supplementary Figure 5. Forest plot for IBS symptom scores of acupuncture versus western medicine at follow-ups; Supplementary Figure 6. Forest plot for quality of life of acupuncture versus nonsham control. a. at the end of treatment; (b) at follow-ups; Supplementary Figure 7. Forest plot for efficacy rate between acupuncture and nonsham acupuncture group-a subgroup analysis; Supplementary Figure 8. Funnel plot for publication bias detection. [file 2871505.f1.zip › 2871505.f1/Supplementary Table 2.doc]

**Supplementary Table 2** Sensitivity analyses

| **Item** | | | **Total studies** | | **Total participants** | **Meta**  **methods** | | | **Results** | |
| --- | --- | --- | --- | --- | --- | --- | --- | --- | --- | --- |
| **Efficacy rates (acupuncture vs. sham acupuncture)** | | | 2 | | 273 | Risk Ratio (M-H, Random, 95% CI) | | | 1.18 [0.92, 1.50] | |
| **Efficacy rates (acupuncture vs. non-sham controls)** | | | 13 | | 1325 | Risk Ratio (M-H, Random, 95% CI) | | | 1.16 [1.09, 1.23] | |
| **Acupuncture vs. western medicine** | | | 10 | | 920 | Risk Ratio (M-H, Random, 95% CI) | | | 1.17 [1.08, 1.27] | |
| **Acupuncture vs. Chinese medications** | | | 2 | | 220 | Risk Ratio (M-H, Random, 95% CI) | | | 1.11 [0.89, 1.38] | |
| **Acupuncture vs. Moxibustion** | | 2 | | | 185 | Risk Ratio (M-H, Random, 95% CI) | | 1.20 [1.04, 1.39] | | |
| **Symptoms** | 11 | | | 1070 | Std. Mean Difference (IV, Random, 95% CI) | | -0.78 [-1.21, -0.35] | | |  |
| **Acupuncture vs. western medicine** | 9 | | | 790 | Std. Mean Difference (IV, Random, 95% CI) | | -0.98 [-1.53, -0.42] | | |  |
| **Acupuncture vs. Chinese medications** | 2 | | | 220 | Std. Mean Difference (IV, Random, 95% CI) | | -0.06 [-0.33, 0.20] | | |  |
| **Acupuncture vs. Moxibustion** | 1 | | | 60 | Std. Mean Difference (IV, Random, 95% CI) | | -0.48  [-1.00, 0.03] | | |  |
| **Acupuncture vs. Antispasmodics on efficacy rates** | 9 | | | 627 | Risk Ratio (M-H, Random, 95% CI) | | 1.21 [1.12, 1.31] | | |  |
| **Acupuncture vs. Antispasmodics on symptoms** | 6 | | | 412 | Std. Mean Difference (IV, Random, 95% CI) | | -1.66 [-2.49, -0.84] | | |  |
